# A New C-Type Lectin Homolog SpCTL6 Exerting Immunoprotective Effect and Regulatory Role in Mud Crab *Scylla paramamosain*

**DOI:** 10.3389/fimmu.2021.661823

**Published:** 2021-04-09

**Authors:** Wanlei Qiu, Fangyi Chen, Roushi Chen, Shuang Li, Xuewu Zhu, Ming Xiong, Ke-Jian Wang

**Affiliations:** ^1^ State Key Laboratory of Marine Environmental Science, College of Ocean & Earth Sciences, Xiamen University, Xiamen, China; ^2^ State-Province Joint Engineering Laboratory of Marine Bioproducts and Technology, College of Ocean & Earth Sciences, Xiamen University, Xiamen, China; ^3^ Fujian Innovation Research Institute for Marine Biological Antimicrobial Peptide Industrial Technology, College of Ocean & Earth Sciences, Xiamen University, Xiamen, China

**Keywords:** C-type lectin, SpCTL6, development, immunoprotective effect, regulatory role

## Abstract

C-type lectin (CTL), a well-known immune-related molecule, has received more and more attention due to its diverse functions, especially its important role in development and host defense of vertebrate and invertebrate. Since the research on crab CTLs is still lack, we screened a new CTL homolog, named SpCTL6 from mud crab *Scylla paramamosain*. The full-length cDNA sequence of SpCTL6 was 738 bp with a 486 bp of ORF, and the deduced amino acids were 161 aa. SpCTL6 was predicted to have a 17 aa signal peptide and its mature peptide was 144 aa (MW 16.7 kDa) with pI value of 5.22. It had typical CTL structural characteristics, such as a single C-type lectin-like domain, 4 conserved cysteines, similar tertiary structure to that of vertebrate CTLs and a mutated Ca^2+^ binding motif Gln-Pro-Thr (QPT), clustering into the same branch as the crustacean CTLs. SpCTL6 was highly expressed in the entire zoeal larval stages and widely distributed in adult crab tissues with the highest transcription level in testis. During the molting process of juvenile crabs, the expression level of SpCTL6 was remarkably increased after molting. SpCTL6 could be significantly upregulated in two larval stages (Z1 and megalopa) and adult crab testis under immune challenges. Recombinant SpCTL6 (rSpCTL6) was successfully obtained from eukaryotic expression system. rSpCTL6 exhibited binding activity with PAMPs (LPS, lipoteichoic acid, peptidoglycan, and glucan) and had a broad spectrum bacterial agglutination activity in a Ca^2+^-dependent manner. In addition, rSpCTL6 could enhance the encapsulation activity of hemocytes and has no cytotoxic effect on hemocytes. Although rSpCTL6 had no bactericidal activity on *Vibrio alginolyticus*, rSpCTL6 treatment could significantly reduce the bacterial endotoxin level *in vitro* and greatly improved the survival of *S. paramamosain* under *V. alginolyticus* infection *in vivo*. The immunoprotective effect of rSpCTL6 might be due to the regulatory role of rSpCTL6 in immune-related genes and immunological parameters. Our study provides new information for understanding the immune defense of mud crabs and would facilitate the development of effective strategies for mud crab aquaculture disease control.

## Introduction

Mud crabs, *Scylla paramamosain*, due to their deliciousness, rich nutrition and high commodity value, have become an important global economic fishery species, which are mainly distributed in the Western Indo-Pacific (1). The capture fishery of mud crabs has been unable to meet the market demand, and the mud crab aquaculture industry has emerged and developed rapidly (2, 3). In 2018, the aquaculture production of mud crabs in China (157,712 tons) far exceeded that of the captured ones (79,444 tons), making it the most productive crab among marine aquaculture crabs in China (4). Although the total production of mud crabs has increased significantly in recent years, the artificial breeding technology of mud crab seedlings is still one of the main scientific and technological problems that have not been solved yet. High mortality occurs frequently during the developmental stage, especially the metamorphosis stage of the larvae [the survival rate was only about 10% from hatching to first-stage crabs (5)], the molting stage of juvenile crabs, and the reproductive molting process, which is closely related to the vulnerability of *S. paramamosain* to pathogens in these processes (6–8). In the past few decades, the research on mud crab immune system has made considerable progress, and many efforts have been made to encourage the development of mud crab aquaculture, such as the use of probiotics, immune-stimulants, and natural products, as reviewed in-depth previously (6). However, these strategies are limited, and we are still far away from understanding the molecular mechanisms of mud crab immunity.

C-type lectin (CTL), a well-known immune-related molecule, has received more and more attention due to its diverse functions, especially its important role in development and host defense of vertebrate and invertebrate (9, 10). Having at least one CTL-like domain (CTLD) is an indispensable feature of the CTLs superfamily (11). Most reported CTLs showed Ca^2+^-dependent carbohydrate binding activity and were considered as pattern recognition receptors (PRRs), with a few exceptions (9). Since the first CTL was discovered in bovine in 1906, more than a thousand CTL members have been identified in Metazoa, with dozens or even hundreds of CTL homologous genes in their genomes (10, 12). In vertebrate, these highly diverse CTLs have been demonstrated to play an important role in the regulation of innate and adaptive immunity, through non-self-recognizing or mediating intercellular communication and interaction, and could act as effector molecules exhibiting directly antibacterial activity (9, 13, 14). And CTLs with single CTLD in invertebrate are also well-studied, with insects and crustaceans as representatives (10). Similarly, the immune function of CTLs in invertebrates is evolutionarily conserved. They participate in both cellular and humoral immunity of invertebrates, such as promoting hemocyte phagocytosis, mediating hemocyte encapsulation, nodule formation, activating the phenol oxidase (PO) system and ultimately leading to melanization, direct bactericidal activity, regulating immune signal transduction pathways and the expression of immune effectors etc., as summarized in detail previously (10, 15, 16).

Currently, several crustacean genomes have been sequenced and assembled, such as the Pacific white shrimp *Litopenaeus vannamei (* 17), the marbled crayfish *Procambarus virginalis (* 18), the Chinese mitten crab *Eriocheir sinensis (* 19), and the swimming crab *Portunus trituberculatus (* 20), which would greatly facilitate the research on crustacean CTLs. So far, more than 90 crustacean CTLs have been reported. Among them, significant progress has been made in the study of shrimp CTLs, whose immune-related functions, molecular mechanisms involved in host-virus (WSSV) interactions, and the expression regulatory mechanism have been thoroughly discussed, providing a valuable reference for the research on other crustacean CTLs (15, 16). In crabs, numerous studies in CTLs have also been conducted, such as the *E. sinensis* CTLs (21–26), and the *P. trituberculatus* CTLs (27–30).

For the mud crab *S. paramamosain*, a total of six CTLs (SpCTLs) have been reported, including SpLec1 and SpLec2 (31), Sp-lectin3 and Sp-lectin4 (32), SpCTL-B (33), SpCTL5 (34). Analysis of SpCTLs coding sequences showed that they all contained only one CTLD, and four of them had signal peptides (SpLec1, SpLec2, Sp-lectin3 and Sp-lectin4), which is common in most crustacean CTLs. Conserved carbohydrate recognition motifs have also been found in SpCTLs, such as QPD (Gln-Pro-Asp) in Sp-lectin4 (32), EPD (Glu-Pro-Asp) in SpCTL-B (33). All SpCTLs were predominantly distributed in the hepatopancreas of adult mud crabs, which is an important immune organ. Among them, SpLec1 and SpLec2, Splectin3 and Sp-lectin4 were also distributed in the embryo and larval stage of crabs, indicating that they might play an important role in the development of crabs. Immune stimulations [such as *Vibrio parahaemolyticus*, *Vibrio alginolyticus*, LPS, Poly (I:C)] could significantly induce the expression of SpCTLs. Recombinant SpCTL protein expression was only performed in SpCTL-B (rSpCTL-B) and SpCTL5 (rSpCTL5) using prokaryotic expression system. *In vitro* functional studies showed that both rSpCTL-B and rSp-CTL5 showed bacterial agglutination activity in a Ca^2+^-dependent manner, while only rSpCTL-B exhibited potent antibacterial activity. When the expression of SpCTL-B was inhibited by RNAi, the bacterial load in hemocytes increased significantly, and several immune-related genes [including SpSTAT and five antimicrobial peptides (AMPs)] were down-regulated, indicating its role in the immune response and regulation (33). Although some progress has been made in *S. paramamosain* CTLs, the functions and molecular mechanisms of SpCTLs are still largely unknown. Therefore, more SpCTLs are expected to be screened and more in-depth studies are needed.

In this study, a new CTL homolog, named SpCTL6 was identified from mud crab *S. paramamosain*. The full-length cDNA sequence was obtained. The expression profiles of SpCTL6 gene was analyzed by absolute quantitative PCR (qPCR) and relative qPCR. The recombinant protein (rSpCTL6) was expressed in *Pichia pastoris* eukaryotic expression system and purified by affinity chromatography. The binding, hemagglutination, agglutination, and encapsulation activity analysis of rSpCTL6 were performed *in vitro*. In addition, the cytotoxicity and the endotoxin level of *V. alginolyticus* after rSpCTL6 treatment were determined. The immunoprotective effect and the regulatory role of rSpCTL6 in *S. paramamosain* challenged with *V. alginolyticus* were evaluated through analyzing the survival rate of crabs, the bacterial clearance ability in gills and hepatopancreas, the expression of immune-related genes and the enzymatic activity of immunological parameters including phenol oxidase (PO), lysozyme (LZM), peroxidase (POD), superoxide dismutase (SOD), alkaline phosphatase (AKP), and acid phosphatase (ACP). This study aims to enrich the knowledge of crab CTLs through in-depth study of its function, immune protective effect and related mechanism, providing important information for understanding the immune defense of mud crabs and facilitating the development of effective strategies for mud crab aquaculture disease control.

## Materials and Methods

### Animals, Sample Collection and Immune Challenge

Adult male and female mud crabs (*S. paramamosain*) weighing about 300 ± 30 g were purchased from a crab farm in Zhangzhou City, Fujian Province, China. The hemocytes were prepared as previously described (35). Multiple tissues of crabs (n=5) were sampled (including gills, hepatopancreas, midgut, eyestalks, subcuticular epidermis, heart, muscle, stomach, thoracic ganglion, female crab gonadal tissues (ovaries, spermatheca, and reproductive duct) and male crab gonadal tissues (testis, anterior vas deferens, seminal vesicle, posterior vas deferens, ejaculation ducts, posterior ejaculation ducts) and ready for total RNA extraction. The various stages of crab development, including embryonic stages (Em1-Em5), zoeal larval stages (Z1-Z5), megalopa stage, three juvenile stages (JU1, JU2, and JU3), the molting process of juvenile crabs including premolt and postmolt samples (JU1 developed to JU2, JU2 developed to JU3) (n=5) were collected from a crab breeding farm in Beihai City, Guangxi Province, China. The different larval stages have their unique features, which can be easily identified as described in detail previously (36, 37).

Two larval stages (Z1 larvae and megalopa) were selected for immune challenge experiments. Thousands of Z1 larvae or megalopa were randomly divided into 6 petri dishes (1.5 L), and 700 mL of sterilized sea water was added. The experiment included three different groups: the control group (sea water), the bacterial infection group (5×10^6^ colony-forming units (CFU)/mL *V. alginolyticus*), and the LPS stimulation group [200 ng/mL LPS (Sigma, USA)]. The larvae were sampled at 3 h, 6 h, 9 h, 12 h, and 24 h after challenge. Five biological parallel samples (n=5) were set up at each time point (each parallel sample contained dozens of larvae). They were immediately put into liquid nitrogen and then stored at -80°C for later use.

The purchased adult male crabs (300 ± 30 g) were acclimated at 23 ± 2 °C for several days before the challenge experiment. Ninety crabs were randomly divided into three groups (30 crabs for each group), including the control group, the *V. alginolyticus* infection group (3×10^6^ CFU/crab), and the LPS stimulation group (0.5 mg/kg·crab). For the control group, 100 μL of crab saline (4.96 mM NaCl, 9.52 mM KCl, 0.8 mM MgSO_4_, 16.2 mM CaCl_2_, 0.84 mM MgCl_2_, 5.95 mM NaHCO_3_, 20 mM HEPES, pH 7.4) was injected. Testis (n=5) were sampled in each group at 3, 6, 12, 24, 48 and 72 h post-injection (hpi) and then stored in -80 °C for later use.

Total RNA of the collected samples were extracted using Trizol reagent (Thermo Fisher Scientific, USA) according to the manufacturer′s instructions. RNA quality was evaluated by NanoDrop 2000 spectrophotometer (Thermo Fisher Scientific, USA), and PrimeScript™ RT reagent kit with gDNA eraser (TaKaRa, Japan) was used for cDNA synthesis.

### Cloning of the Full-Length cDNA Sequence of SpCTL6

The cDNA of adult male crab testis was synthesized as described above. The 5′ and 3′ RACE cDNA were prepared using SMARTer^®^ RACE 5′/3′ Kit (Clontech, USA). The coding sequence (CDS) of SpCTL6 was obtained from the transcriptome database established by our laboratory. A pair of primers (SpCTL6-CDS-F and SpCTL6-CDS-R, as shown in **Table 1**) were designed to amplify SpCTL6 CDS and testis cDNA was used as a template. The PCR was carried out as follows: 95°C, 5 min; 30 cycles of 95°C, 30 s, 62°C, 30 s, 72°C 1 min; 72°C for 5 minute. And primers (SpCTL6-5-R1, SpCTL6-5-R2, SpCTL6-3-F1, SpCTL6-3-F2, as shown in **Table 1**) were synthesized to amplify the 5′ and 3′ cDNA ends of SpCTL6 using nested PCR and touchdown PCR. The first round of PCR used pairs of primers (SpCTL6-5-R1/Long primer for 5′ RACE PCR, SpCTL6-3-F1/Long primer for 3′ RACE PCR) and the second round of PCR used SpCTL6-5-R2/Short primer for 5′ RACE PCR, SpCTL6-3-F2/Short primer for 3′ RACE PCR, respectively. The Long primer and Short primer were provided by SMARTer^®^ RACE 5′/3′ Kit. The touch down PCR procedure was performed as followed: 95°C, 5 min; 30 cycles of 95°C, 30 s, 68°C -0.5/cycle, 30 s, 72°C, 2 min; 72°C, 10 min; 16°C 5 min. The PCR products were then purified and sequenced by Sangon Biotech (Shanghai) Co., Ltd.

**Table 1:** Sequences of primers used in this study.

Primer name	Primer sequences (5′-3′)
SpCTL6-CDS-F	ATGCTGCGCGTGTACTGCCTCCTCC
SpCTL6-CDS-R	TCAGAAGGCGTGGACCTCGTTCTGA
SpCTL6-5-R1	AGCCACGTGGTTGTAATCGAA
SpCTL6-5-R2	AAGCACATGTAGTTGAGCACG
SpCTL6-3-F1	TTGACGACCGTGCCCTTAG
SpCTL6-3-F2	GCCCTTAGCCCCAACTCTATT
Long primer	CTAATACGACTCACTATAGGGCAAGCAGTGGTATCAACGCAGAGT
Short primer	CTAATACGACTCACTATAGGGC
SpCTL6-qPCR-F	ACGACGCCTCCTGGTTTTGG
SpCTL6-qPCR-R	GGCGTGGACCTCGTTCTGAC
SpSOD-F	GGGGATGGGAAACAACTCTGGAT
SpSOD-R	GGTGCCTTGGTTAAATACACGGTGC
SpALF6-F	TCAAGGGAGACGTGTGGTGC
SpALF6-R	TGGCGAAGTCTGCGATAGCC
SpCrustin3-F	ACCTGCCTGGCCATTACGTG
SpCrustin3-R	CCCACCACAGGGAGTGTTGC
GADPH-qPCR-F	CTCCACTGGTGCCGCTAAGGCTGTA
GADPH-qPCR-R	CAAGTCAGGTCAACCACGGACACAT
rSpCTL6-*EcoR*I-F	CGGAATTCGCGTGCCCTGCCCCCTTTGT
rSpCTL6-*Not*I-R	ATAAGAATGCGGCCGCTCA**GTGGTGGTGGTGGTGGTG**GAAGGCGTGGACCTCGTTC

### Bioinformatics and Phylogenetic Analysis of SpCTL6

The homology of the SpCTL6 gene sequence with other sequences was analyzed by the basic local alignment search tool (BLAST) of NCBI (http://www.ncbi.nlm.nih.gov/blast). The signal peptide and conserved domain of SpCTL6 were predicted by SignalP 4.1 Server (http://www.cbs.dtu.dk/services/SignalP/) and SMART database (http://smart.embl-heidelberg.de/), respectively. Expasy (http://web.expasy.org/protparam) was used to calculate the theoretical molecular weight (MW) and isoelectric point (pI) of SpCTL6. The secondary and tertiary structures of SpCTL6 were predicted by UCL (http://bioinf.cs.ucl.ac.uk/psipred/) and SWISS-MODEL (https://swissmodel.expasy.org/), respectively. Multiple sequence alignment between SpCTL6 and other CTLs was performed using Clustal X 2.1 software. The phylogenetic tree was constructed using the neighbor joining method of MEGA 6.0, and the reliability was evaluated by 1000 bootstraps.

### Quantitative Real-Time PCR Analysis of the Expression Profiles of SpCTL6

The expression profiles of SpCTL6 gene in various adult crab tissues and different developmental stages were determined by absolute quantitative real-time PCR (qPCR) and the expression changes of SpCTL6 during the molting stages of juvenile crabs and the response patterns of SpCTL6 gene to LPS and *V. alginolyticus* challenge were analyzed by relative qPCR. GAPDH gene of *S. paramamosain* (GenBank accession number: JX268543.1) was employed as the internal reference gene in relative qPCR assay. Gene-specific primers (SpCTL6-qPCR-F/SpCTL6-qPCR-R, GADPH-qPCR-F/GADPH-qPCR-R, listed in **Table 1**) were designed. The SpCTL6 CDS plasmid was used to generate its standard curve. qPCR was performed on Qtower 2.2 (Analytik Jena, Germany) as followed: 50 °C for 2 min, 95 °C for 10 min, 40 cycles of 95 °C for 15 s, 60 °C for 1 min. The absolute copy numbers of SpCTL6 gene were calculated according to the linear regression of the standard curve. The data for relative qPCR was analyzed using the algorithm of the 2^-ΔΔCt^ method (38).

### Expression and Purification of Recombinant SpCTL6 (rSpCTL6) in *Pichia Pastoris* Eukaryotic Expression System

The mature peptide sequence of SpCTL6 was constructed into the pPIC9K vector using primers rSpCTL6-*EcoR*I-F/rSpCTL6-*Not*I-R (as shown in **Table 1**). The recombinant plasmid was linearized by restriction enzyme *Sac* I, and then transformed into competent *P. pastoris* cells by electroporation. A positive clone was picked and cultured to logarithmic growth phase at 28 °C in YPD medium (2% tryptone, 1% yeast extract, 2% D-glucose). Then the medium was replaced with BMGY medium (10 g yeast extract, 20 g tryptone were dissolved in 700mL water, autoclaved for 20 min, cooled to room temperature, and the following mixture were added: 100 mL 1 M potassium phosphate buffer (pH 6.0), 100 mL 10 YNB, 100 mL10% glycerine, 2 mL 500×biotin) until it cultured to logarithmic then *P. pastoris* cells were induced by 0.5% methanol in BMMY (dissolve 10 g yeast extract, 20 g tryptone in 700 mL water, autoclaviate for 20 min, cool to room temperature, add the following mixture: 100 mL 1 M potassium phosphate buffer (pH 6.0), 100 mL 10 YNB, 100 mL10% methanol, 2 mL 500×biotin). After 24 hours of induction, the supernatant was collected and dialyzed against buffer (50 mM phosphate buffer, 50 mM NaCl, pH 9.0). The target protein was purified by ÄKTA Pure system (GE Healthcare Life Sciences, USA) using a HisTrap™ FF Crude column (GE Healthcare Life Sciences, USA). The purity of rSpCTL6 was analyzed by SDS-PAGE, and the sequence was confirmed by the Mass Spectrometry Center of the School of Life Sciences, Xiamen University. The protein concentration was determined by Bradford assay kit (Beyotime Institute of Biotechnology, China). All recombinant protein rSpCTL6 were stored at -80°C for later use.

### Hemagglutination, Bacterial Agglutination, Binding, and Encapsulation Assay

#### Hemagglutination Assay

The blood of mouse was collected in a centrifuge tube containing sodium citrate. After washing 3 times with TBS buffer, the blood was re-suspended in TBS buffer at a concentration of 2% (v/v), and then transferred to a 96-well type V hemagglutination plate. Four groups were set up (rSpCTL6 with or without 10 mM CaCl_2_ groups, TBS with 10 mM CaCl_2_ group, and BSA with10 mM CaCl_2_ group). rSpCTL6 was added at a series of dilution concentrations (from 0.75 μg/mL to 100 μg/mL). An equal volume of BSA (100 μg/mL) and TBS with 10 mM CaCl_2_ were added as control groups. Hemagglutination was observed after incubation at 4°C for 1 h. Each group had three biological parallels. The independent experiment was repeated at least three times.

#### Bacterial Agglutination Assay

Gram-positive bacteria (*Staphylococcus aureus*), and Gram-negative bacteria (*Pseudomonas aeruginosa*, *Pseudomonas fluorescens*, *Aeromonas hydrophila*, *Vibrio harveyi*, *Vibrio fluvialis*, *V. parahaemolyticus*, and *V. alginolyticus*) were selected to investigate the agglutination activity of rSpCTL6. All the bacteria were cultured to the logarithmic growth phase, washed 3 times with TBS, and re-suspended in 0.1 M NaHCO_3_ (pH 9.0). They were then incubated with a final concentration of 0.1 mg/mL FITC (Sigma, USA) for 30 min at room temperature, and washed 3 times with TBS to remove unlabeled FITC. The concentration of the bacteria was adjusted to 10^8^ CFU/mL. Four groups were set up (rSpCTL6 with or without 10 mM CaCl_2_ groups, TBS with 10 mM CaCl_2_ group, and BSA with10 mM CaCl_2_ group). The FITC-labeled bacteria were incubated with 10 μL of rSpCTL6 (40 μg/mL) at room temperature in the dark for 1 h. An equal volume of BSA (40 μg/mL) and TBS with 10 mM CaCl_2_ were added as control groups. The agglutination results were observed with an inverted fluorescence microscope (Zeiss, Germany). Each group had three biological parallels. The independent experiment was repeated at least three times.

#### Binding Assay

A modified enzyme-linked immune sorbent assay (ELISA) was carried out as previously described (39). Briefly, LPS, peptidoglycan (PGN), lipoteichoic acid (LTA) and glucan (GLU) were diluted with an ELISA coating solution to a working concentration of 20 μg/mL, and then added to a 96-well ELISA plate. The plate was coated overnight at 4°C, blocked with 5% skim milk at 37°C for 2 h, and then added with a series dilution of rSpCTL6 (0~100 μg/mL) at 37°C for 2 h. There were 3 parallels for each concentration of protein. Mouse anti-His antibody (1:5000, prepared in 1% skim milk) was added and incubated at 37 °C for 2 h followed by incubation with goat anti-mouse HRP antibody (1:5000). TMB solution was added and incubated at 37 °C for 10-30 min. The reaction was stopped with 2 M H_2_SO_4_. The absorbance at 450 nm was measured using a microplate reader (TECAN GENios, GMI, USA). Each group had three biological parallels. The independent experiment was repeated at least three times.

#### Encapsulation Assay

In order to evaluate the encapsulation activity of rSpCTL6, the Ni-NTA agarose beads [Senhui Microsphere Technology (Suzhou) Co., Ltd, China] was used. Four groups were also set up (rSpCTL6 with or without 10 mM CaCl_2_ groups, TBS with 10 mM CaCl_2_ group, and BSA with10 mM CaCl_2_ group). The beads were first equilibrated in TBS, then incubated with different amounts of rSpCTL6 (25 μg-200 μg) at 4 °C overnight, and washed with TBS 3 times. A 24-well culture plate was pre-coated with 1% agarose. The hemocytes were isolated from *S. paramamosain* as previously described (35), and added to the plate for at least 10 min to allow them settle down. The beads containing proteins were then added and incubated at 26 °C. Encapsulation was detected after 6 and 24 h under a light microscope (LEICA DMi1, Germany). Each group had three biological parallels. The independent experiment was repeated at least three times.

### *V. Alginolyticus* Endotoxin Assay

The endotoxin level of *V. alginolyticus* was detected by the Toxin Sensor™ Chromogenic LAL Endotoxin Assay Kit (GenScript, USA) following the manufacturer′s instructions. *V. alginolyticus* was collected when reaching to the logarithmic growth phase and adjusted to a concentration of 10^7^ CFU/mL. They were then incubated with different concentration of rSpCTL6 (10.5, 21, 42, 84 µM) at room temperature for 1 h and analyzed by a spectrophotometer at an absorbance of 545 nm (Agilent Technologies. Malaysia). Each concentration of rSpCTL6 treatment had three biological parallels. The independent experiment was repeated at least three times.

### Cytotoxicity Assay

The hemocytes of adult crabs were primarily cultured as previously described (40, 41). About 1×10^5^ cells/well were inoculated on a 96-well cell culture plate at 26 °C and cultured overnight. Different concentration of rSpCTL6 (3 μM, 6 μM, 12 μM, 24 μM, 48 μM) were added. After 24 h of incubation, the CellTiter 96^®^ AQueous Kit (Promega) was used to evaluate the viability of hemocytes. Each concentration of rSpCTL6 treatment had three biological parallels. The independent experiment was repeated at least three times.

### Effect of rSpCTL6 on Mud Crab *S. Paramamosain* Infected by *V. Alginolyticus*

#### Mortality Test

To investigate whether rSpCTL6 has an immunoprotective effect on *S. paramamosain*, a bacterial challenge experiments was performed in male mud crabs (100 ± 10 g). *V. alginolyticus* (2 × 10^7^ CFU crab^-1^) were first injected into the base of the right fourth leg of the crabs (n=105). After bacterial injection, 105 crabs were randomly divided into three groups (n=35) (control group, 10 μg crab^-1^ rSpCTL6 treatment group and 20 μg crab^-1^ rSpCTL6 treatment group). Two hours later, rSpCTL6 (10 μg crab^-1^ or 20 μg crab^-1^) was injected, and an equal volume of the crab saline was injected as the control group. Survival crabs were recorded at different time point (3 h, 6 h, 9 h, 24 h, 36 h, 48 h, 60 h, 72 h, 96 h, 120 h), and a mortality curve was drawn by GraphPad Prism 8.3.0 version. The independent experiment was repeated twice.

#### Bacterial Load Assay

The bacterial load in two crab tissues (gills and hepatopancreas) of different groups (control group and 20 μg crab^-1^ rSpCTL6 treatment group) was determined at selected time points after treatment (3 h, 6 h, and 24 h). Briefly, gills and hepatopancreas (n=3) were sampled from experimental crabs (treated with crab saline or 20 μg crab^-1^ rSpCTL6 after 2 h of bacterial injection), and approximately 100 mg of each tissue was homogenized in 1 mL of PBS solution, then diluted to an appropriate concentration, and spread on an agar plate [containing 2216E cultured medium (Qingdao Hope Bio Tech., China)], and cultured at 28 °C overnight. The bacterial CFU of all plates were counted and recorded. Each sample had three biological parallels and three different dilutions.

#### Enzymatic Activity Assay

The gills and hepatopancreas were sampled (n=3) and homogenized as described above and various enzymatic activity assays were performed according to the manufacturer′s instructions of the corresponding protease detection kits [including PO, lysozyme (LZM), peroxidase (POD), superoxide dismutase (SOD), alkaline phosphatase (AKP), acid phosphatase (ACP) assays (Nanjing Jiancheng, China)].

#### Immune-Related Genes Expression Analysis

Several immune-related genes were selected to study their expression pattern after treatment (including signaling pathway genes (*Sp*Toll2, Relish, *Sp*Dorsal, STAT), AMPs [*Sp*Crustin3, *Sp*Crustin5, *Sp*ALF2, *Sp*ALF6), cytokine gene (LITAF), antioxidant gene (*Sp*SOD)]. The specific primers and genbank accession numbers for these genes were summarized in **Tables 1** and **S1**. Relative qPCR was performed as described above.

### Statistical Analysis

GraphPad Prism 8.0 Software was applied for analyzing relative qPCR assay and enzymatic activity assay using multiple t test - one per row. The mortality curve was drawn by GraphPad Prism 8.0 Software and analyzed using the Kaplan-Meier Log rank test. Difference was considered as significant at *p* < 0.05. All data were displayed as mean ± standard deviation (SD).

## Results

### Sequence and Structure Analysis of SpCTL6

**Figure 1:**
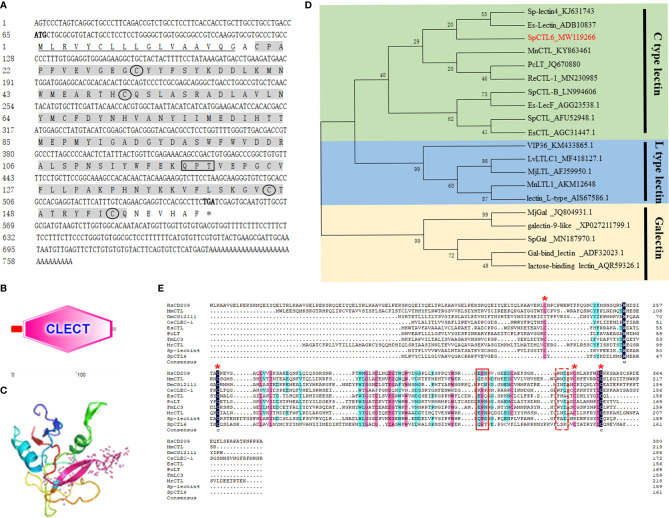
Bioinformatics and phylogenetic analysis of SpCTL6. **(A)** The nucleotide sequence and deduced amino sequence of SpCTL6 from *Scylla paramamosain*. Start codon (ATG) and stop codon(TGA) are marked in bold. The signal peptide is underlined and the CTLD is shade with gray. Four conserved residues are circled and the carbohydrate binding motif (QPT) is boxed. **(B)** Schematic representation of the structural domain of SpCTL6 protein predicted by SMART. **(C)** A predicted three-dimensional model of SpCTL6 by SWISS-MODEL program. **(D)** Phylogenetic tree analysis of SpCTL6. A neighbor-joining phylogenetic tree of lectins was constructed using MEGA ver.6.0 software with 1000 bootstrap replications. SpCTL6 was shown in red. **(E)** Multiple sequence alignment of CTLs from various species. Amino acids residues that are completely conserved are shaded in dark blue, and similar amino acids are shaded in red or light blue. Four conserved cysteine residues involved in the formation of the disulfide bridges are marked with red asterisks, the carbohydrate binding motif (QPT) is boxed with solid line and the WND motif is boxed with dot line. The information of the sequences are listed as follows: HsCD209 (*Homo sapiens*, AAI10616), MmCTL (*Mus musculus* AAD05125), DmCG12111 (*Drosophila melanogaster*, AAF46391), CeCLEC-2 (*C. elegans*, CCD61973), EsCTL (*E. sinensis*, JX841199), PcLT (*Procambarus clarkia*, JQ670880), FmLC3 (*Fenneropenaeus merguiensis*, JF430082), MrCTL (*Macrobrachium rosenbergii*, KX495215), Sp-lectin4 (*S. paramamosain*, KJ631743), SpCTL6 (*S. paramamosain*, MW119266).

The full-length cDNA sequence of SpCTL6 was 738 bp with a 486 bp of ORF, and the deduced amino acids was 161 aa (**Figure 1A**). SpCTL6 was predicted to have a 17 aa signal peptide and its mature peptide was 144 aa (MW 16.7 kDa) with pI value of 5.22, which contained a conserved CTLD (**Figures 1A, B**). Its tertiary structures were predicted, which has a double-loop structure, 2 α-helices and 5 β-sheets (**Figure 1C**). The phylogenetic analysis showed that SpCTL6 was clustered into the same branch as the crustacean CTLs (**Figure 1D**). Multiple sequences alignment analysis showed that it has 4 conserved cysteines and a Gln-Pro-Thr (QPT) motif (**Figure 1E**).

### The Expression Profiles of SpCTL6

**Figure 2:**
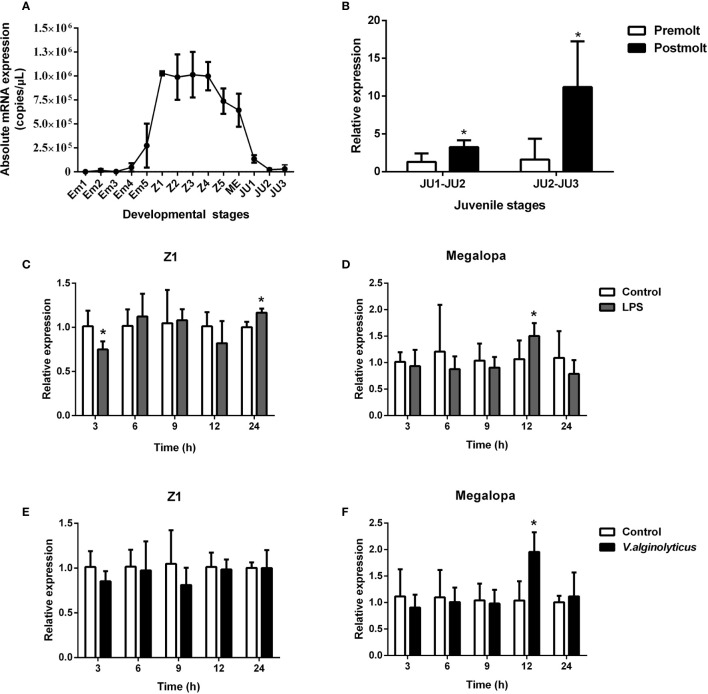
Expression profiles of SpCTL6 gene in the developmental stages of *S. paramamosain*. **(A)** The expression of SpCTL6 in the different developmental stages of *S. paramamosain* was determined by absolute qPCR (n=5). Em1-Em5: embryonic stage 1-5; Z1-Z5: zoeal larval stage 1-5; ME: megalopa; JU: juvenile crab. **(B)** The expression of SpCTL6 in molting process of juvenile crabs was analyzed by relative qPCR (n=5); The expression pattern of SpCTL6 in Z1 (n=5) and megalopa (n=5) after LPS **(C, D)** and *V. alginolyticus* **(E, F)** challenges was analyzed by relative qPCR. Significant difference was indicated by asterisks as *p < 0.05.

Based on qPCR analysis, SpCTL6 mRNA was at a relatively low level in the embryonic stage (Em1-Em4) of *S. paramamosain*. Its copy numbers gradually increased from the Em5 stage, reached the highest levels in the Z1 stage (about 1×10^6^ copies/μL), maintained a high level (over 0.7×10^6^ copies/μL) throughout the zoea larval stage, and then gradually reduced from the megalopa stage to juvenile crabs (**Figure 2A**). During the molting process of juvenile crabs, the expression of SpCTL6 was significantly upregulated after molting (JU1 developed to JU2, and JU2 developed to JU3) (**Figure 2B**). As reported, high mortality occurs frequently during the developmental stages, especially the metamorphosis stage of the larvae (5). Among them, Z1 is the larvae that has just hatched from embryo, and megalopa is the last larval stage undergoing metamorphosis from zoeal stage, which are two important metamorphosis stages. Therefore, we chose these two stages as representatives to investigate the immune response of SpCTL6 in the larval stages. After LPS stimulation, in the Z1 stage of *S. paramamosain*, SpCTL6 was significantly down-regulated at 3 h and up-regulated at 24 h, while the expression of SpCTL6 did not show any change under the challenge of *V. alginolyticus* (**Figures 2C, E**). In the megalopa stage, the transcription level of SpCTL6 mRNA was remarkably increased at 12 h after LPS or bacterial challenge (**Figures 2D, F**).

**Figure 3:**
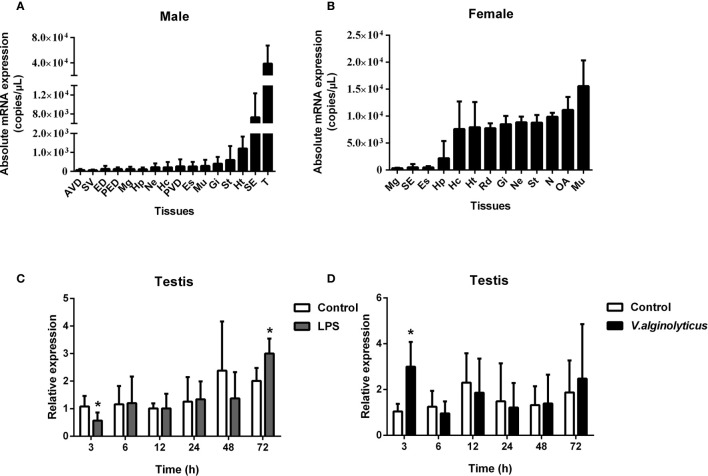
Expression profiles of SpCTL6 gene in *S. paramamosain* adult. Tissue distribution of SpCTL6 in male **(A)** and female **(B)** crabs (n=5). The expression pattern of SpCTL6 in male testis after LPS **(C)** and *V. alginolyticus* **(D)** challenges (n=5). Significant difference was indicated by asterisks as *p < 0.05. AVD, anterior vas deferens; SV, seminal vesicle; ED, ejaculatory duct; PED, posterior ejaculatory duct; Mg, midgut; Hp, hepatopancreas; Ne, thoracic ganglion; Hc, hemocytes; PVD, posterior vas deferens; Es, eye stalk; Mu, muscle; Gi, gills; St, stomach; Ht, heart; SE, subcuticular; T, testis; RD, reproductive duct; N: spermathecae; OA, ovaries.

The absolute qPCR results also showed that SpCTL6 was widely distributed in various tissues of male and female adult crabs, with the highest expression level in the testis of male adult crabs (**Figures 3A, B**). When adult male crabs were injected with LPS or *V. alginolyticus*, the expression of SpCTL6 was significantly down-regulated at 3 h and up-regulated at 72 h after LPS stimulation, and up-regulated at 3 h after bacterial challenge (**Figures 3C, D**).

### Recombinant SpCTL6 (rSpCTL6) Obtained From *P. pastoris* Eukaryotic Expression System

**Figure 4:**
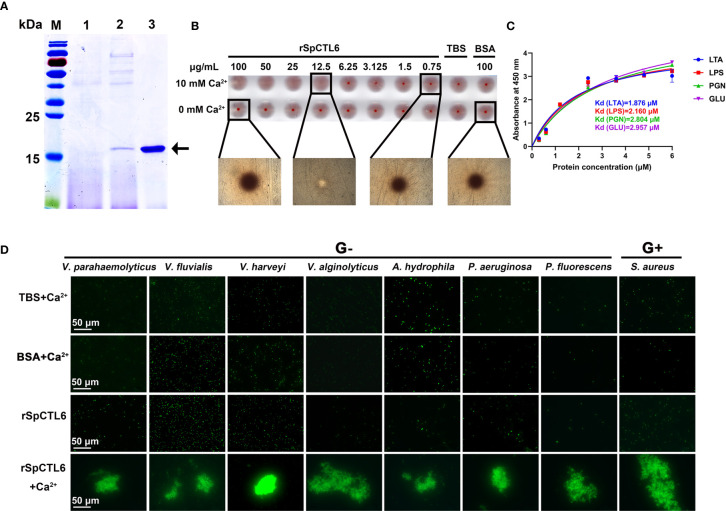
Analysis of hemagglutination, agglutination and binding activity of rSpCTL6. **(A)** Expression and purification of recombinant SpCTL6. Lane M: protein molecular standard; lane 1: negative control for rSpCTL6 without methanol induction; lane 2: expression of rSpCTL6 after methanol induction; lane 3: purified rSpCTL6. **(B)** Agglutination activity of rSpCTL6 on mouse erythrocytes. **(C)** Binding activity of rSpCTL6 to PAMPs (LTA for lipoteichoic acid, LPS for lipopolysaccharide, PGN for peptidoglycan, GLU for glucan). **(D)** Bacterial agglutinating activity of rSpCTL6 on FITC-labeled Gram-negative bacteria (G^-^) *V. parahaemolyticus*, *V. fluvialis*, *V. harveyi*, *V. alginolyticus*, *A hydrophila*, *P. aeruginosa*, *P. fluorescens*, and Gram-positive bacteria (G^+^) *S. aureus*. The concentration of CaCl_2_ was 10 mM. TBS and BSA were used as control groups.

We successfully obtained recombinant SpCTL6 (rSpCTL6) from the *P. pastoris* eukaryotic expression system. SDS-PAGE analysis showed that a specific positive band appeared at a position slightly larger than the 15 kDa marker, corresponding to the predicted mature peptide size (16.7 kDa), and the purity of rSpCTL6 was high as shown in **Figure 4A**. In addition, the results from the mass spectrometry also confirmed that the purified protein was the target protein rSpCTL6 (**Figure S1**).

### rSpCTL6 Showed Hemagglutination, Bacterial Agglutination, Binding, and Encapsulation Activity *In Vitro*

For the type V hemagglutination plate used in hemagglutination assay, if the erythrocytes are observed to settle at the bottom of the plate, it indicates that the reaction is negative, that is, the erythrocytes have not agglutinated. The results showed that in the presence of 10 mM Ca^2+^, rSpCTL6 at a low concentration of 12.5 μg/mL could cause significant agglutination of mouse erythrocytes, not to mention higher concentrations (25, 50, and 100 μg/mL) (**Figure 4B**). In the 0 mM Ca^2+^ group and the control group, obvious sedimentation of erythrocytes was observed (**Figure 4B**). Therefore, the hemagglutination activity of rSpCTL6 was Ca^2+^ dependent. Similarly, rSpCTL6 could agglutinate all the selected bacteria (including several Vibrio species commonly found in aquaculture) in a Ca^2+^-dependent manner (**Figure 4D**). While the control groups (TBS and BSA) and rSpCTL6 without CaCl_2_ group could not induce agglutination of tested bacteria.

As shown in **Figure 4C**, rSpCTL6 had binding affinity to several tested microbial surface molecules (LPS, LTA, PGN, and GLU). The calculated apparent dissociation constants (Kd) were 1.876 μM for LTA, 2.160 μM for LPS, 2.804 μM for PGN, 2.957 μM for GLU, respectively, which indicated that rSpCTL6 had the highest binding affinity for LTA.

**Figure 5:**
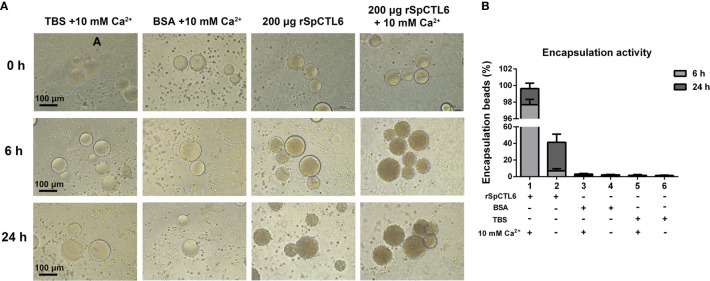
Analysis of encapsulation activity of rSpCTL6. The agrose beads pre-coated with rSpCTL6 were observed for encapsulation by crab hemocytes at 0, 6 and 24 h post-incubation **(A)**. The beads coated with TBS and BSA were used as control groups. **(B)** The percentage of beads encapsulated by hemocytes after 6 h and 24 h of incubation.

rSpCTL6 pre-coated with Ni-NTA agarose beads was used to perform the encapsulation assay *in vitro*. The results showed that there was no obvious hemocytes encapsulating around the beads in the control groups and the encapsulation ratio was very low (TBS and BSA, with or without Ca^2+^ group) (**Figures 5A, B**). While in 200 μg rSpCTL6 treatment group, after 6 h of incubation, dozens of hemocytes were recruited around the beads, the color of the beads became light dark, and the encapsulation ratio was about 7% (**Figures 5A, B**). Especially, in the presence of 10 mM Ca^2+^, 200 μg rSpCTL6 treatment for 6 h resulted in an encapsulation ratio of about 98% and the beads color became black dark (**Figures 5A, B**). The encapsulation ratio of 24 h rSpCTL6 treatment without or with Ca^2+^ reached about 40% and 99.6%, respectively (**Figures 5A, B**). And the encapsulation activity of rSpCTL6 exhibited in a dose-dependent manner (**Figure S2**).

### rSpCTL6 Had No Cytotoxicity on Hemocytes of *S. Paramamosain* and Could Reduce the *V. Alginolyticus* Endotoxin Level *In Vitro*

**Figure 6:**
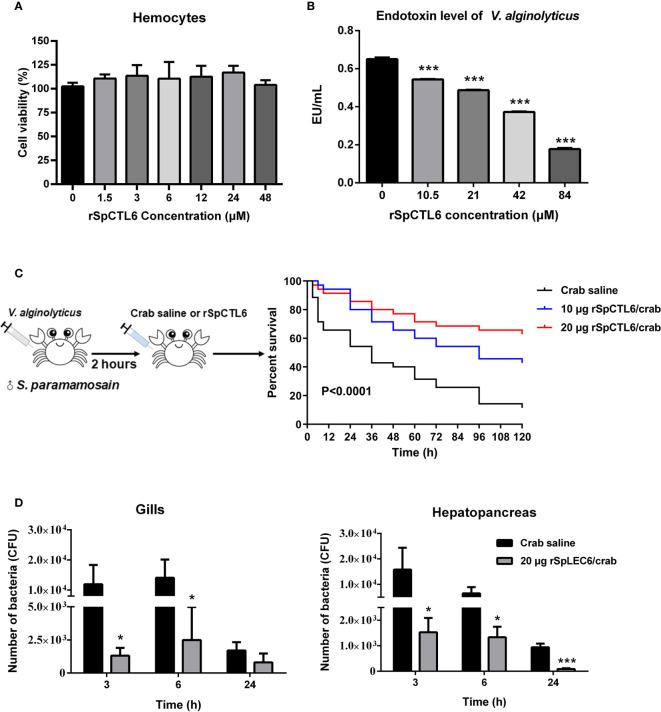
Evaluation of cytotoxicity and endotoxin level of *V. alginolyticus* after rSpCTL6 treatment *in vitro* and the immunoprotective effect of rSpCTL6 in *S. paramamosain*. Cytotoxic effect of rSpCTL6 on crab hemocytes **(A)** and endotoxin level of *V. alginolyticus* after rSpCTL6 treatment *in vitro* **(B)**. The survival curve of mud crabs after rSpCTL6 treatment **(C)**. Male crabs were challenged with *V. alginolyticus*, and SpCTL6 was injected (10 μg crab^-1^ or 20 μg crab^-1^) at 2 h post bacterial challenge (n=35). Bacterial clearance of *V. alginolyticus* in the gills and hepatopancreas **(D)**. The bacterial burden in the gills and hepatopancreas were determined at 3, 6 and 24 hpi. * represented *p < *0.05 and *** represented *p* < 0.001.

The cytotoxicity of rSpCTL6 on hemocytes of *S. paramamosain* was evaluated. The results showed that different concentrations of rSpCTL6 (from 1.5 μM to 48 μM) had no cytotoxic effect on the primarily cultured crab hemocytes (**Figure 6A**), which would provide evidence for the safety use of this protein in subsequent *in vivo* study.

In addition, it was found that low concentration rSpCTL6 treatment (10.5 μM) could significantly reduce the endotoxin level of *V. alginolyticus*, which also showed a dose-dependent manner, that is, higher protein treatment concentration, lower bacterial endotoxin level (**Figure 6B**). And under the treatment of 84 μM rSpCTL6, the endotoxin level was reduced by about 70% (**Figure 6B**).

### The Immunoprotective Effect of rSpCTL6 on Mud Crab *S. Paramamosain* Challenged with *V. Alginolyticus*

In order to investigate the immunoprotective effect of rSpCTL6 and its regulatory role on the immune system of mud crab *S. paramamosain* under bacterial infection, a series of assays were conducted, including mortality test, bacterial load assay, enzymatic activity assay, and immune-related genes expression analysis.

The schematic diagram of the bacterial challenge and treatment process was shown in **Figure 6C**. The mud crab mortality curve was drawn (**Figure 6C**). The results showed that in the crab saline group, the survival rate dropped sharply in the early stage, and reached about 54% survival rate after 24 h of treatment. While for the rSpCTL6 treatment groups (10 μg crab^-1^ or 20 μg crab^-1^), the survival rate at 24 h was 80% and 85.7%, respectively (*P*<0.0001) (**Figure 6C**). 120 h after treatment, the survival rate of the crab saline group was only 11%, while 42.8% for the 10 μg rSpCTL6 crab^-1^ treatment group and 62.9% for the 20 μg rSpCTL6 crab^-1^ treatment group (*P*<0.0001) (**Figure 6C**). These results indicated that rSpCTL6 treatment could significantly increase the survival of mud crab *S. paramamosain* infected with *V. alginolyticus*.

The bacterial loads in gills and hepatopancreas of *S. paramamosain* after bacterial infection and different treatments were determined. As shown in **Figure 6D**, rSpCTL6 treatment at different time points (3 h, 6 h, and 24 h) could significantly reduce the bacterial number in gills and hepatopancreas of crabs. For example, 3 h after treatment, the bacterial counts in gills and hepatopancreas of the control group were around 1.19×10^4^ CFU and 1.58×10^4^ CFU, while the corresponding bacterial counts in rSpCTL6 treatment group decreased to 1.32×10^3^ CFU, 1.53×10^3^ CFU, respectively. With the treatment time increased (from 3 h to 24 h), the amount of bacteria gradually decreased, no matter in the control group or the treatment group (**Figure 6D**).

### The Regulatory Role of rSpCTL6 on Mud Crab *S. Paramamosain* Infected by *V. Alginolyticus*

**Figure 7:**
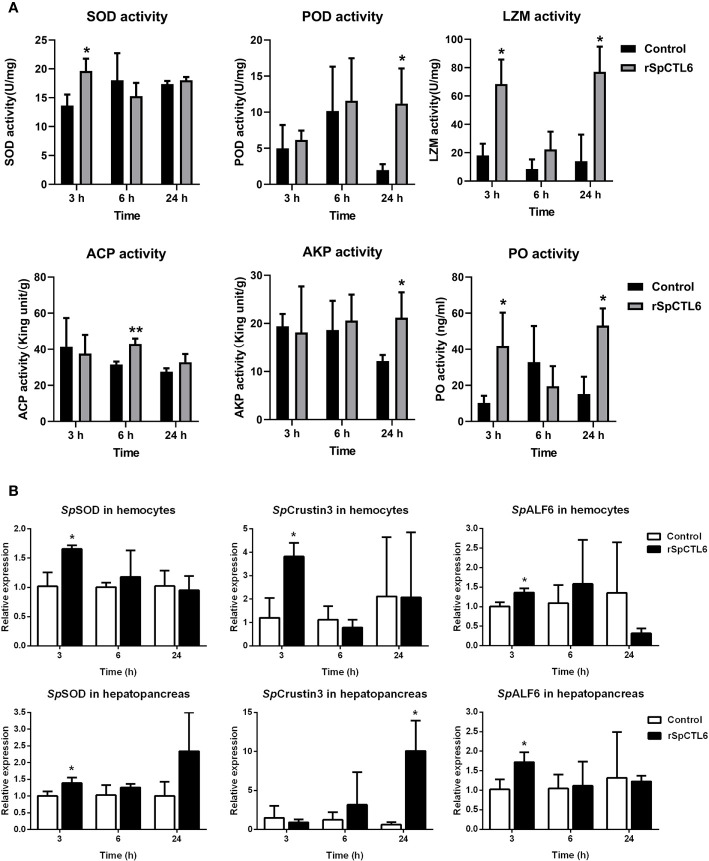
The regulatory role of rSpCTL6 in *S. paramamosain*. Enzymatic activity analysis (SOD, POD, LZM, ACP, AKP, and PO) in hepatopancreas after rSpCTL6 treatment of *S. paramamosain* challenged with *V. alginolyticus* (n=3) **(A)**. The immune-related gene (*Sp*SOD, *Sp*Crustin3 and *Sp*ALF6) expression in hemocytes and hepatopancreas were examined (n=3) **(B)**. Significant difference between rSpCTL6 treatment group and the crab saline injection group was indicated by asterisks as *p < 0.05.

In order to further investigate the molecular mechanism of the immunoprotective effect of rSpCTL6, we analyzed the activity of several immunological parameters and the expression of genes involved in mud crab immunity. The results showed that the activity of two antioxidant enzymes SOD and POD was significantly upregulated at 3 h and 24 h after rSpCTL6 treatment, respectively (**Figure 7A**). For ACP and AKP, their response time was also different, whose activity was remarkably increased at 6 h and 24 h after rSpCTL6 treatment, respectively (**Figure 7A**). In addition, both LZM and PO activity were found to have significant enhancement at two time points (3 h and 24 h after rSpCTL6 treatment) (**Figure 7A**).

We randomly chose several typical immune-related genes to analyze their gene expression modulations in hemocytes and hepatopancreas of *S. paramamosain* after rSpCTL6 treatment. The results showed that among them, three genes (including *Sp*SOD, *Sp*Crustin3 and *Sp*ALF6) were significantly upregulated in both hemocytes and hepatopancreas at 3 h after rSpCTL6 treatment, with *Sp*Crustin3 upregulated at 24 h in hepatopancreas (**Figure 7B**). While the expression of other selected genes showed no changes, except for *Sp*ALF2 down-regulated at 24 h in hemocytes (**Figure S3**).

## Discussion

Although the genome of *S. paramamosain* is not available, cDNA library construction and transcriptome analysis has become a powerful tool to identify functional genes (35, 42–44). Since the research on SpCTLs is still lack, we screened a new CTL homolog, named SpCTL6 from the embryonic and larval developmental transcriptome database established in our laboratory. It was found that the transcription level of this newly selected CTL gene was relatively high in the entire Zoeal larval stages, and gradually decreased when it developed into megalopa stage and juvenile crabs, which attracted our attention to investigate its function. Subsequent verification by qPCR also confirmed the reliability of the transcriptome data, indicating that SpCTL6 might play important roles in the development of zoeal larval stages. We then cloned the full-length cDNA of SpCTL6 and determined its expression profiles. In addition, we obtained the recombinant protein (rSpCTL6) from eukaryotic expression system and the immune-related function of rSpCTL6 *in vitro* and *in vivo* were elucidated.

Most reported crustacean CTLs have signal peptides, as well as SpCTL6 in this study, indicating that they might belong to secreted expression proteins and act as PRRs, opsonins or effectors to help eliminate invading pathogens (10). In vertebrates, CTL has 4 highly conserved cysteines (Cys), which form disulfide bridges to stabilize its double-loop tertiary structure with 2 α-helices and 5 β-sheets (11). Besides, there are 4 Ca^2+^-binding sites and site 2 has been confirmed to participate in carbohydrate binding through two typical motifs [EPN (Glu-Pro-Asn) or QPD (Gln-Pro-Asp), and WND (Trp-Asn- Asp)] (11). However, since there is no report on crystal structure analysis of crustacean CTLs, we could only obtain information from their structural prediction and sequence alignment with other CTLs. It is found that crustacean CTLs are diverse, in which those conserved motifs in vertebrate are usually mutated, such as EPK (Glu-Pro-Lys), EPS (Glu-Pro-Ser), EPQ (Glu -Pro-Gln), QPG (Gln-Pro-Gly), QPS (Gln-Pro-Ser), QPN (Gln-Pro-Asn), QPT (Gln-Pro-Thr), as summarized in detail in shrimp CTLs (15). Among SpCTLs, only Sp-lectin4 has a typical QPD motif (32), while SpCTL-B contains EPD (Glu-Pro-Asp) motif (33), which is not found in SpLec1, SpLec2, Sp-lectin3 and SpCTL5 (31, 32, 34). For SpCTL6 in the study, although its amino acid identity is relatively low compared with other crustacean CTLs, it had typical CTL structural characteristics, such as a single CTLD, 4 conserved Cys, similar tertiary structure to that of vertebrate CTLs and the mutated Ca^2+^ binding motif (QPT), clustering into the same branch as the crustacean CTLs, which provided evidence that SpCTL6 is a novel CTL homolog.

In recent years, a large number of studies have focused on the immune function of CTLs, but the role of CTLs in development cannot be ignored. At present, relevant researches have been carried out on insects. It was reported that a CTL HaCTL3 from the cotton bollworm *Helicoverpa armigera* played an important role in larval growth and development through modulating ecdysone and juvenile hormone signaling (45). Knock-down the expression of Tcctl5 led to developmental defects of *Tribolium castaneum*, such as reduced survival rate in early adults, and loss of locomotor activity in adults (46). The schlaff (slf) gene coding a putative C-type lectin was necessary for the adhesion between the horizontal cuticle layers of Drosophila (47). So far, research on the function of crustacean CTLs in the development are few and not in-depth. In *S. paramamosain*, it was found that SpLec1 and SpLec2 had certain expression in the zoeal stages of *S. paramamosain*, while Sp-lectin3 and Sp-lectin4 were widely distributed in embryos, larvae and crablet stages (31, 32). *V. parahaemolyticus* challenge could up-regulated the expression of SpLec1 and SpLec2 in megalopa stage of *S. paramamosain*, indicating their role in development and immune response (31). In the study, we systematically analyzed the expression of SpCTL6 in the entire embryonic (Em1-5) and larval stages (Z1-Z5, megalopa) and three stages of juvenile crabs (JU1-3). The absolute copy number of SpCTL6 during zoeal stages reached 0.7~1×10^6^ copies/μL. Such a high transcription level indicated that SpCTL6 might be involved in the modulation of zoeal stage development. However, whether the development-related signaling pathways (such as ecdysone and juvenile hormone signaling) could also be modulated by SpCTL6 similar to HaCTL3 requires further investigation. The upregulated expression pattern of SpCTL6 in Z1 and megalopa stages under immune challenges further demonstrated that SpCTL6 might exert dual roles (developmental modulation and immune response) in the early development of *S. paramamosain*. In addition, during the molting process of juvenile crabs, the expression SpCTL6 was significantly upregulated after molting (JU1 developed to JU2, and JU2 developed to JU3). Since crabs are vulnerable to infection by foreign microorganisms during molting, they might adopt certain strategies, such as increasing the expression of immune factors (including SpCTL6) to resist pathogens. Though the underlying molecular mechanism is still unclear, the results obtained in this study provide new insights into the role of crustacean CTLs in development.

The currently reported crustacean CTLs are widely distributed in various tissues, most of which are predominantly expressed in the hepatopancreas, and some CTLs mainly distributed in hemocytes and gills, or other tissues (heart, gills, intestine, stomach, brain) (15, 21, 30, 33, 34). However, there are few reports on the specific high expression of CTLs in the gonads of crustaceans. Interestingly, SpCTL6 was highly expressed in the testis of adult male crabs (its absolute copy number was the highest compared with other male tissues and all female tissues), which is quite different from reported crustacean CTLs. In other aquatic invertebrates, it was found that two CTLs, AjCTL-1 from sea cucumber *Apostichopus japonicus* and AiCTL-9 from scallop *Argopecten irradians* also had the highest expression level in gonad (48, 49). Wang et al. performed a genome-wide survey on the lectin domain containing proteins (LDCPs) of oyster *Crassostrea gigas* and found that C-type lectin domain containing proteins (CTLPs) were the most abundant proteins (50). Among them, 40 CTLPs were highly expressed in male gonad, indicating their important role in the reproductive immunity (50). Similar screening methods can also be used in the discovery of crustacean CTLs, which would help us further understand the function of this superfamily. Due to the lack of model animals, it is relatively difficult to investigate the molecular mechanism of functional genes in aquatic invertebrates. In the model organism *Caenorhabditis elegans*, a CTLP IRG-7 was demonstrated to regulate the immune response of reproductive systems of *C. elegans* to defense against pathogens (51). In *S. paramomosain*, several male-specific immune effectors antimicrobial peptides (AMPs) have been identified in our previous studies, including scygonadin (52–54), SCY2 (55), and scyreprocin (39). All of them have been revealed to participate in the reproductive immunity of mud crabs. In the present study, SpCTL6 was found to be significantly regulated in the testis under LPS or *V. alginolyticus* challenge, indicating that it participated in the immune response of *S. paramamosain*. Therefore, we speculated that the high expression of SpCTL6 in male gonad might also be involved in mud crab reproductive immunity, which will be further studied in the future.

Most functional studies of crustacean CTLs have been conducted through obtaining the recombinant proteins and analyzing the *in vitro* activity (including antimicrobial, binding, agglutination, and encapsulation activities etc.) (10, 15). Prokaryotic expression system is widely used in the recombinant expression of crustacean CTL proteins, and the vectors included pET-32a, pET-21a, and pGEX-4T-1 etc. (33, 34, 56). As we all know, the yeast eukaryotic expression system is also a commonly used protein expression system, which can undergo mRNA splicing and post-translational modifications for eukaryotic organisms, thereby would better mimic the existing form of the protein *in vivo * (57). In particular, the correct spatial structure and modification are very important for protein function. In this study, we obtained high-purity recombinant SpCTL6 (rSpCTL6) from *P. pastoris* eukaryotic expression system, and confirmed it using mass spectrometry, which provides a critical guarantee for our further functional study.

As PRRs, crustacean CTLs exhibited strong binding activity with pathogen associated molecular patterns (PAMPs, such as LPS, LTA, PGN and GLU) and bacteria, some of which had direct bactericidal activity. In swimming crab *P. trituberculatus* and Chinese mitten crab *E. sinensis*, several CTLs showed both binding activity with PAMPs and bacteria, such as PtCLec1, PtCTL-2, PtCTL-3, EsCTL1, EsCTL2, and EsCTLDcp (22, 23, 27, 30). SpCTL-B in *S. paramamosain* could bind to a variety of bacteria, including Gram-positive bacteria *S. aureus* and *Streptococcus hemolyticus*, Gram-negative bacteria *E. coli*, *V. parahaemolyticus*, *V. alginolyticus*, *A. hydrophila* and fungi *Saccharomyces cerevisiae *(33). Several crab CTLs showed potent antibacterial activity, such as SpCTL-B (33), PtCLec1 (30), rEsLecH (21), EsLecA and EsLecG (25). For rSpCTL6, it had binding activity with 4 PAMPs 9LPS, LTA, PGN, and GLU) with moderate binding affinity [“moderate” was defined as Kd value of 100 nM to 10 μM) (58)]. However, rSpCTL6 could not bind to bacteria and had no obvious antimicrobial activity (data not shown), indicating that it might exert immune functions through other molecular mechanisms.

Agglutination activity is also the hallmark biological function of CTLs. By recognizing molecules on the surface of bacteria, CTLs can directly immobilize pathogens and agglutinate them, so that pathogens will not spread in the body. A large number of studies have shown that most CTLs rely on calcium ions to exert or enhance their agglutination activity (10). Crab CTLs exhibited a broad spectrum of bacterial agglutination activity in Ca^2+^-dependent manner, especially on several typical aquaculture pathogens, such as *V. alginolyticus*, *V. parahaemolyticus*, *V. vulnificus*, and *A. hydrophila *(22, 23, 27, 30, 33). Among them, EsLecA, EsLecG and EsLecD agglutinated *E. coli* and *P. pastoris* without relying on calcium ions, and the addition of Ca^2+^ could significantly enhance their agglutinating activity (25, 59). In addition, PtCLec1, PtCTL4 and PtCTL1 had hemagglutination activity on rabbit erythrocytes in the presence of Ca^2+^ (28–30). Similar broad spectrum bacterial agglutination activity was also found in rSpCTL6, which could agglutinate 5 common pathogens in aquaculture (*A. hydrophila*, *V. harveyi*, *V. fluvialis*, *V. parahaemolyticus*, and *V. alginolyticus*), and rSpCTL6 had hemagglutination activity on mouse erythrocytes in a Ca^2+^-dependent manner.

When some large pathogens such as parasites invade the crustacean, the host hemocytes will wrap around them, forming a multiple cell layers, so that those pathogens cannot move freely in the host, and then they will be killed by melanin or endotoxin released by hemocytes (60). Therefore, hemocyte encapsulation ability play an important role in cellular immunity of invertebrates (61). Studies have found that crustacean CTLs can enhance the ability of hemocytes to encapsulate large pathogens and then followed by melanization (15). Agarose beads are usually used as invading objects to evaluate the effect of CTLs on the encapsulation activity of hemocytes. When the agarose beads were incubated with PtCTL-2 and PtCTL-3 from *P. trituberculatus* separately, and then the hemocytes were added, the hemocytes encapsulated 57% and 45% of agarose beads respectively, while the control group had no obvious coating effect (27). In *E. sinensis*, EsLecA, EsLecG, EsLecD and EsLecF could also activate the encapsulation activity of hemocytes on agarose beads (25, 59, 62). The results showed that after 24 h of incubation, almost 100% of EsLecD-coated agarose beads were encapsulated by hemocytes, which might be due to the high binding affinity of EsLecD to hemocyte surface molecules (59). Similarly, in our study, rSpCTL6 significantly enhanced the hemocyte encapsulation on agarose beads and might cause hemocyte melanization to help eliminate invading pathogens, indicating its important role in cellular immunity of *S. paramamosain*.

Research on the function of recombinant CTLs *in vitro* is far from enough, and elucidating the immune protective and regulatory effects of CTL proteins *in vivo* will provide important evidences for further understanding their immune functions. Most of the reported crustacean CTLs conducted the *in vivo* bacterial clearance assay to evaluate the *in vivo* effects of recombinant CTLs. They first pre-incubated the bacteria and recombinant CTL protein for a period of time, and then co-injected them into animals, and finally counted the numbers of living bacteria in the hemolymph. The results showed that this treatment could remarkably accelerate the bacteria clearance, such as rPtCLec1 (30), EsCTL1 and EsCTL2 (22), EsCTLDcp (23) in crab, PcLec3 (63), PcLec4 (64), MnCTL (56), MrCTL (65), LvCTL3 (66), FmLC4 (67), and FcLec4 (68) in crayfish, prawn and shrimp. Whether CTLs can significantly improve the survival rate of animals under pathogenic infection is an important evidence to clarify their immune protective effects. PcLec4 overexpression *in vivo* exhibited obviously higher survival rate (50% at the ninth day) than that of the control groups (10% at the ninth day) (64). Pre-incubated rPcLT and WSSV could effectively protect crayfish *Procambarus clarkii* from WSSV infection, with a much higher survival rate (around 75%) than that of the control groups (3-5%) (69). Injection of WSSV or *V. alginolyticus* together with rLvCTL3 could significantly reduce the mortality of *L. vannamei* infected by WSSV and *V. alginolyticus *(66). In our study, the treatment method was different from those reports mentioned above. We first infected the crabs with *V. alginolyticus* and 2 hours later, injected rSpCTL6 or crab saline into the crabs. We would like to know the *in vivo* protective effect of rSpCTL6 under pathogenic infection. The results showed that rSpCTL6 treatment could also enhance the bacterial clearance in gills and hepatopancreas. In addition, rSpCTL6 treatment could greatly improve the survival of infected crabs [increased by about 50% (62.9% in rSpCTL6 treatment group, 11% in the control group in 120h)], which would provide a valuable information for the disease control in mud crab aquaculture.

At present, the regulatory role of crustacean CTLs in the immune system has been extensively studied mainly through the use of RNA interference (RNAi), which is a powerful tool for revealing the molecular mechanism of functional genes (15). In crabs, such as in *E. sinensis*, RNAi of EsLecH could down-regulate the expression of several AMPs (including Es-DWD1, EsALF, and EsALF-2) and phosphorylation of JNK (21). When the mRNA level of JNK was inhibited, the addition of rEsLecH could not cause changes in AMP expression, indicating that EsLecH required JNK signaling to perform its regulation function (21). Knock down of PtCLec1 from *P. trituberculatus* could regulate the expression of genes involved in phagocytosis, proPO system, complement system, Toll and IMD pathways, and AMPs, indicating its key regulatory role in the innate immunity of *P. trituberculatus *(30). In rPcLT treatment shrimp, PO and SOD activity increased significantly at different time points (69). In *S. paramamosain*, several AMPs (SpHistin, SpALF4, SpALF5 and SpALF6), spSTAT and hemocyte phagocytosis were all regulated by SpCTL-B (also evaluated through RNAi), suggesting that SpCTL-B played important roles both in the humoral and cellular immunity of mud crabs (33). In order to understand the underlying molecular mechanism of the immunoprotective effect of SpCTL6, we further analyzed the expression of immune-related genes and several immunological parameters after rSpCTL6 treatment. We found that the expression of two AMP genes SpCrustin3 and SpALF6, an antioxidant gene SOD, and all the tested parameters (including SOD, POD, LZM, ACP, AKP, and PO activity) had been upregulated, indicating the regulatory role of rSpALF6 in the innate immunity of *S. paramamosain*. However, the underlying regulatory mechanism of CTLs is complicated and we are still far from understanding it. As shown in a previous study, it was found that the silencing of LvLdlrCTL (a CTL containing a low-density lipoprotein receptor (LDLR) class A domain) from *L. vannamei* down-regulated the expression of 3 AMPs (crustin, ALF3, and PEN3), but 2 other AMPs (PEN2 and PEN4) were upregulated (70). Similarly, in our study, 2 AMPs (*Sp*Crustin3 and *Sp*ALF6) were upregulated at 3 h in the hemocytes, while another AMP *Sp*ALF2 was down-regulated at 24 h in the hemocytes after rSpCTL6 treatment. In the early stage (3 h and 6 h after rSpCTL6 treatment), the bacterial load in crabs was still high (**Figure 6D**), which might require increased enzyme activity and expression of AMPs to resist bacteria. Our results corresponded to this hypothesis, for example, the activities of a variety of immunological parameters in the hepatopancreas (including SOD, LZM, ACP and PO) and several immune-related genes in hemocytes (including *Sp*SOD, *Sp*Crustin3, *Sp*ALF6) were upregulated at 3 h or 6 h after rSpCTL6 treatment, suggesting that rSpCTL6 might activate the innate immune system of *S. paramamosain*, thereby quickly eliminating the invading bacteria. And at the 24 h time point, the number of bacteria decreased to a low level, we speculated that crabs need to adopted anti-inflammatory strategies to help the body recover. Therefore, the downregulation of *Sp*ALF2 might be due to the need for anti-inflammatory response and the maintenance of homeostasis, which requires further in-depth investigation.

In summary, this study characterized a new CTL homolog SpCTL6 from mud crab *S. paramamosain*. SpCTL6 was highly expressed in the entire zoeal larval stages and widely distributed in adult crab tissues with the highest transcription level in testis. It could be significantly upregulated in two larval stages (Z1 and megalopa stages) and adult crab testis under immune challenges. During the molting process of juvenile crabs, the expression level of SpCTL6 was remarkably increased after molting. Recombinant SpCTL6 was successfully obtained from eukaryotic expression system. rSpCTL6 exhibited binding activity with PAMPs and had a broad spectrum of bacterial agglutination activity in a Ca^2+^-dependent manner. rSpCTL6 could enhance the encapsulation activity of hemocytes and it had no cytotoxic effect on hemocytes. Furthermore, rSpCTL6 treatment could significantly reduce the endotoxin level of *V. alginolyticus in vitro* and greatly improved the survival of *S. paramamosain* under bacterial infection. The immunoprotective effect of rSpCTL6 might be due to the regulatory role of rSpALF6 in the immune-related genes and immunological parameters. Our study provides important information for understanding the immune defense of mud crabs and would facilitate the development of effective strategies for mud crab aquaculture disease control.

## Data Availability Statement

The original contributions presented in the study are included in the article/**Supplementary Material**. Further inquiries can be directed to the corresponding author.

## Ethics Statement

The animal study was reviewed and approved by the Laboratory Animal Management and Ethics Committee of Xiamen University.

## Author Contributions

WQ: Data curation, formal analysis, investigation, and methodology. RC, SL, XZ, and MX: Investigation and methodology. FC: Conceptualization, funding acquisition, project administration, supervision; writing–original draft, and writing–review and editing. KW: Funding acquisition, project administration, and writing–review and editing. All authors contributed to the article and approved the submitted version.

## Funding

This study was supported by grants (grant # U1805233/ # 41806162) from the National Natural Science Foundation of China (NSFC), the Fundamental Research Funds from Central Universities (grant # 20720190109), the Fujian Marine Economic Development 395 Subsidy Fund Project (Grant # FJHJF-L-2019-1) from the Fujian Ocean and Fisheries Department and a grant (# 3502Z20203012) from the Xiamen Science and Technology Planning Project.

## Conflict of Interest

The authors declare that the research was conducted in the absence of any commercial or financial relationships that could be construed as a potential conflict of interest.
