# Supplementary material for: A New C-Type Lectin Homolog SpCTL6 Exerting Immunoprotective Effect and Regulatory Role in Mud Crab Scylla paramamosain
Source: Front Immunol. 2021 Apr 9;12:661823. doi: 10.3389/fimmu.2021.661823 (PMC8062930; doi:10.3389/fimmu.2021.661823)
Supplement: Supplementary file 1 [file DataSheet_1.docx]

**Supplementary materials**

**For**

**A new C-type lectin homolog SpCTL6 exerting immunoprotective effect and regulatory role in mud crab *Scylla paramamosain***

Wanlei Qiu^1^, Fangyi Chen^1,2,3*^, Roushi Chen^1^, Shuang Li^1^, Xuewu Zhu^1^, Ming Xiong^1,2,3^, Ke-Jian Wang^1,2,3^

1 State Key Laboratory of Marine Environmental Science, College of Ocean & Earth Sciences, Xiamen University, Xiamen, Fujian, China

2 State-Province Joint Engineering Laboratory of Marine Bioproducts and Technology, College of Ocean & Earth Sciences, Xiamen University, Xiamen, Fujian, China

3 Fujian Innovation Research Institute for Marine Biological Antimicrobial Peptide Industrial Technology, College of Ocean & Earth Sciences, Xiamen University, Xiamen, Fujian, China

*Corresponding author: Fangyi Chen

College of Ocean & Earth Sciences, Xiamen University, Xiamen, Fujian 361102, PR China.

E-mail: [chenfangyi@xmu.edu.cn](mailto:chenfangyi@xmu.edu.cn) (F. Chen)

**Table and Figures**

**Table S1 Sequences of primers used in the study.**

| **Primer name** | **Primer sequences (5′-3′)** | **Genbank accession NO.** |
| --- | --- | --- |
| Relish-F | CGATTTGCCCAACTGCTTCAGTG | MH047674.1 |
| Relish -R | GTCTGAACTCTTCTTGCCACCGT |  |
| STAT-F | CAGCCGACATACTCAGGCAGTTG | KC711050 |
| STAT-R | GTGAGTTGCTGTTCACCCAGCT |  |
| SpDorsal-F | GTTGCGACCCTCAGACAAGA | MH047675.1 |
| SpDorsal-R | CCTGATGCCTGGCTGATAGG |  |
| SpToll2-F | TGTTGCCTTGGGCTGCATTG | SLM84439.1 |
| SpToll2-R | TCGCGTGGGAAGGCATTGTT |  |
| SpALF2-F | CGCGTGTCGATGCTTCTCGT | HM345950.1 |
| SpALF2-F | ACCACACGTCTCCCCTGAAGT |  |
| SpALF6-F | TCAAGGGAGACGTGTGGTGC | LT560377.1 |
| SpALF6-F | TGGCGAAGTCTGCGATAGCC |  |
| SpCrustin3-F | ACCTGCCTGGCCATTACGTG | AUV47160.1 |
| SpCrustin3-R | CCCACCACAGGGAGTGTTGC |  |
| SpCrustin5-F | TAGCGTTCCTCGTGCTGGTG | AUV47159.1 |
| SpCrustin5-R | ACGAATTGGAGGGCAGCGTC |  |
| SpSOD-F | GGGGATGGGAAACAACTCTGGAT | FJ774661.1 |
| SpSOD-R | GGTGCCTTGGTTAAATACACGGTGC |  |
| LITAF-F | AGGCTGGTGCTCCAACAACTGT | MK510015 |
| LITAF-R | TGCAGTGTGGGCAGCTATGTTC |  |


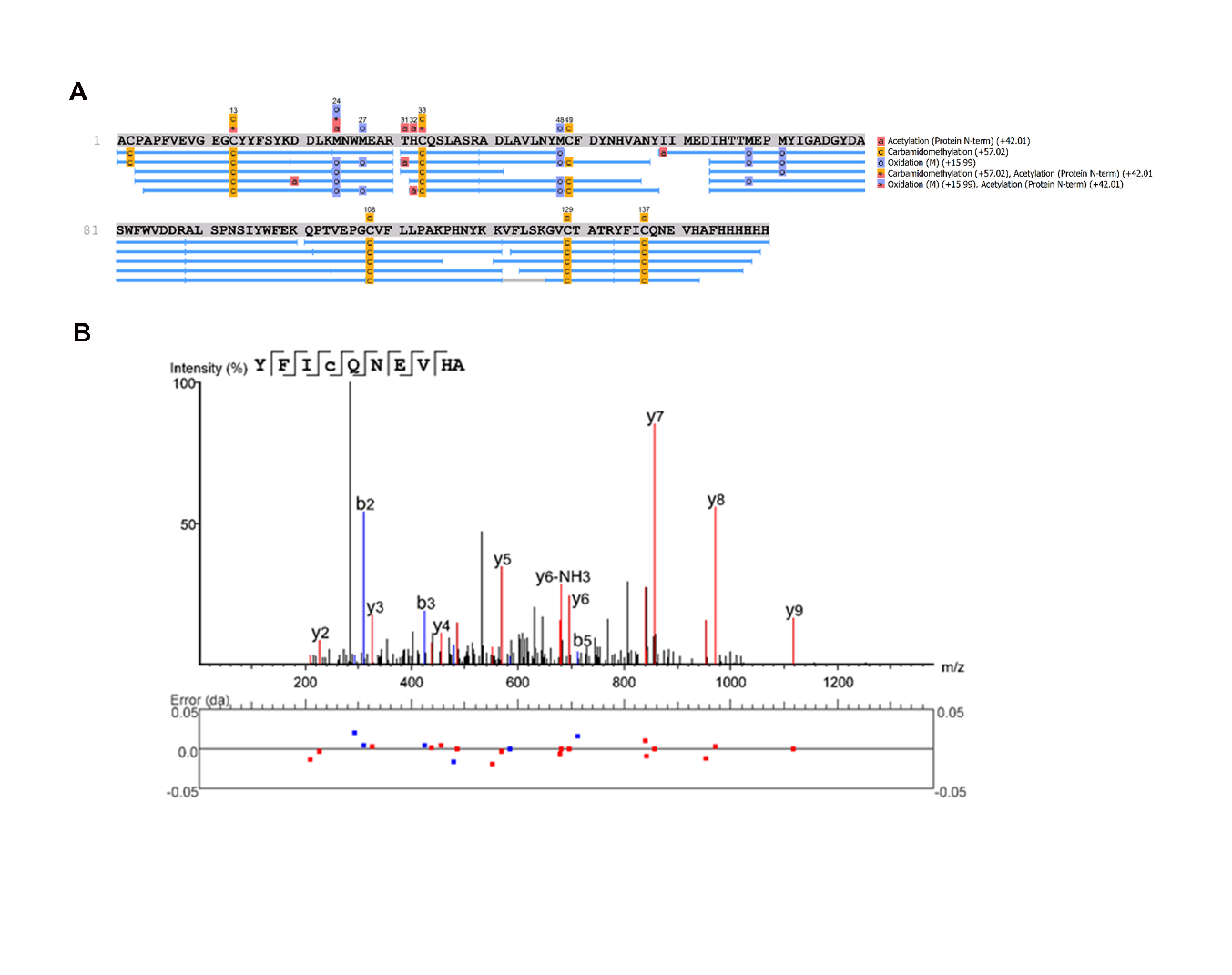
**Fig. S1 Mass spectrometry analysis of rSpCTL6.**


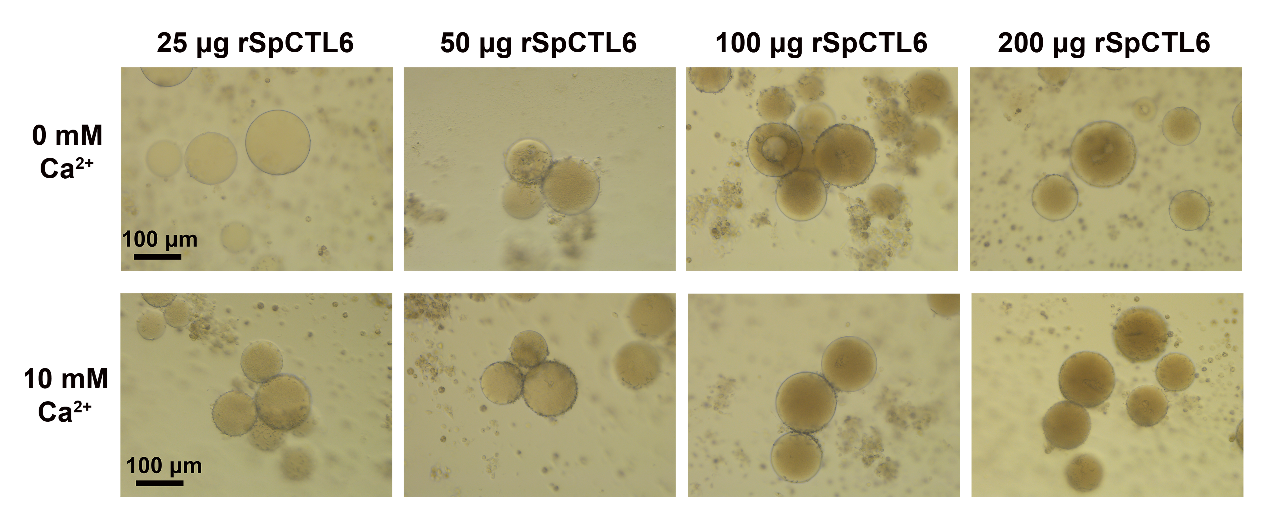


**Fig. S2** **The encapsulation activity of rSpCTL6.** The agrose beads pre-coated with different amount of rSpCTL6 (25 μg-200 μg) were used for detecting hemocyte encapsulation activity at 24 h post-incubation, which showed a dose-dependent manner.


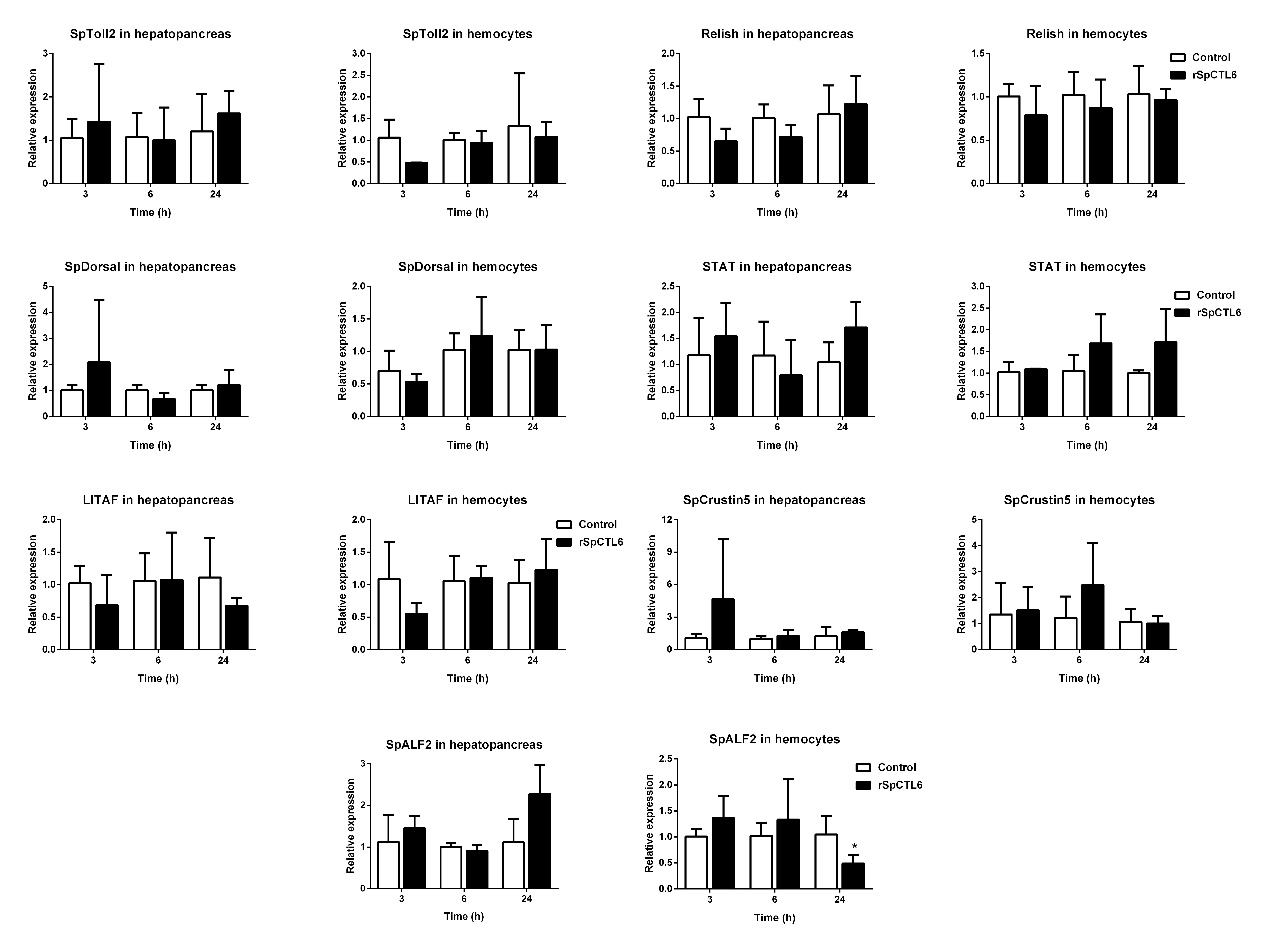


**Fig. S3** **The** **expression of immune-related genes after rSpCTL6 treatment of *S. paramamosain* challenged with *V. alginolyticus.*** The expression of these immune-related genes showed no changes, except for ALF2 down-regulated at 24 h in hemocytes.
